# Evaluation of the Ethiopian Millennium Rural Initiative: Impact on Mortality and Cost-Effectiveness

**DOI:** 10.1371/journal.pone.0079847

**Published:** 2013-11-18

**Authors:** Leslie A. Curry, Patrick Byam, Erika Linnander, Kyeen M. Andersson, Yigeremu Abebe, Abraham Zerihun, Jennifer W. Thompson, Elizabeth H. Bradley

**Affiliations:** 1 Yale School of Public Health and Global Health Leadership Institute, New Haven, Connecticut, United States of America; 2 Futures Institute, Glastonbury, Connecticut, United States of America; 3 Clinton Health Access Initiative, Addis Abba, Ethiopia; Boston Children’s Hospital, United States of America

## Abstract

**Main Objective:**

Few studies have examined the long-term, impact of large-scale interventions to strengthen primary care services for women and children in rural, low-income settings. We evaluated the impact of the Ethiopian Millennium Rural Initiative (EMRI), an 18-month systems-based intervention to improve the performance of 30 primary health care units in rural areas of Ethiopia.

**Methods:**

We assessed the impact of EMRI on maternal and child survival using The Lives Saved Tool (LiST), Demography (DemProj) and AIDS Impact Model (AIM) tools in Spectrum software, inputting monthly data on 6 indicators 1) antenatal coverage (ANC), 2) skilled birth attendance coverage (SBA), 3) post-natal coverage (PNC), 4) HIV testing during ANC, 5) measles vaccination coverage, and 6) pentavalent 3 vaccination coverages. We calculated a cost-benefit ratio of the EMRI program including lives saved during implementation and lives saved during implementation and 5 year follow-up.

**Results:**

A total of 134 lives (all children) were estimated to have been saved due to the EMRI interventions during the 18-month intervention in 30 health centers and their catchment areas, with an estimated additional 852 lives (820 children and 2 adults) saved during the 5-year post-EMRI period. For the 18-month intervention period, EMRI cost $37,313 per life saved ($42,366 per life if evaluation costs are included). Calculated over the 18-month intervention plus 5 years post-intervention, EMRI cost $5,875 per life saved ($6,671 per life if evaluation costs are included). The cost effectiveness of EMRI improves substantially if the performance achieved during the 18 months of the EMRI intervention is sustained for 5 years. Scaling up EMRI to operate for 5 years across the 4 major regions of Ethiopia could save as many as 34,908 lives.

**Significance:**

A systems-based approach to improving primary care in low-income settings can have transformational impact on lives saved and be cost-effective.

## Introduction

Developing high quality and accessible primary care is a global priority [1], [2]. With the global push to meet MDGs 4 and 5 related to maternal and child health, many efforts have been directed at strengthening rural primary care services focused primarily on women and children in low-income settings [3]–[6]. Evidence regarding the impact of various health system strengthening efforts is mixed. Interventions have shown improvements in utilization of and satisfaction with reproductive health services [7], institutional births [8], maternity care including skilled birth attendants [9], [10], emergency obstetric care [5] and antenatal care [4]. Yet other studies have demonstrated mixed results, with little effect on prevention of mother-to-child transmission of HIV [11], and other key outcomes associated with reproductive health [12], maternal and child health [13], [14], and HIV [15].

Despite this literature, few studies have examined the potential long- term impact of interventions on the mortality of women and children in rural, low-income settings, or the sustainability of such interventions. Efforts to study sustainability face multiple impediments, including limited resources (e.g., constraints on project scope, time periods and funding) as well as methodological challenges (e.g., incomplete data, difficulties isolating effects of an intervention from broader secular changes, inability to follow up full sample) [16]. In addition, little is known about cost-effectiveness of health system strengthening interventions to improve primary care. The Millennium Villages study, a multi-sector, multi-country intervention measured current impact of the program and its associated costs, although it did not report overall cost-effectiveness of the intervention [6]. Furthermore, this effort did not focus solely on primary health care; few, if any, studies have examined impact of a large-scale, system-based intervention to improve primary health care in rural settings.

Therefore, we sought to evaluate the impact of the Ethiopian Millennium Rural Initiative (EMRI) in terms of lives saved and cost. EMRI was systems-based intervention to improve the performance of 30 primary health care units (PHCUs) each covering a catchment area of 40,000 people living in rural areas of Ethiopia. The present study builds on our previous work, which described changes in performance of EMRI health centers [17], factors associated with better performance [18], and the key themes related to community partnerships for health in Ethiopia [19]. Findings from this study can be helpful in designing future health systems strengthening efforts aimed at improving primary care in rural settings.

## Methods

### Ethics Statement

All research and consent procedures were approved by the Institutional Review Board at the Yale School of Medicine and the Ethiopian Federal Ministry of Health. Since data was collected in the aggregate from health facilities, no written or verbal informed consent was needed for this study.

### Setting and Intervention

The Ethiopian Millennium Rural Initiative (EMRI) is an 18-month systems-based intervention to improve the performance of 30 primary health care units (PHCUs) in rural areas of Ethiopia. The initiative was designed using mechanisms that, if successful, could be implemented across all health centers nationally by the Ethiopian Federal Ministry of Health (FMOH). The intervention was implemented by the Clinton Health Access Initiative (CHAI), working collaboratively with the FMOH in 4 of the more populous regions of the country: Amhara, Oromia, Tigray and Southern Nations, Nationalities, and Peoples’ Region (SNNPR). EMRI was rolled out in a series of 3 separate and overlapping phases (10 PHCUs in each of Phases I, II and III). Each phase lasted 18 months, with the exception of 5 PHCUs in Phase III, which received only 15 months of intervention due to delays in program startup.

Each PHCU includes a health center (HC) (staffed by approximately 15 health workers including a medical director, nurses, pharmacists, and other health workers), 6 to 7 health posts (HP) (each staffed by 2 health extension workers (HEWs) overseen by a health extension worker supervisor), and a team of approximately 180 volunteer community health workers (VCHWs) who are unpaid but trained and supervised by HEWs to conduct health education and community mobilization activities in the catchment area. Health centers and health posts are owned by the government and overseen by the *woreda* health office, which reports to the Regional Health Bureau (RHB). The *woreda* health office may be co-located in the health center or located within an hour of the health center.

EMRI addressed the following broad areas of PHCU organization: 1) Physical infrastructure, including water, electricity, condition of buildings, and equipment, 2) Human resources capacity, including training and community health worker engagement, 3) Services, including prevention of mother-to-child transmission of HIV (PMTCT) programs, HIV testing during directly observed therapy for tuberculosis (TB DOTS), HIV testing at the health post level, antenatal care, postnatal care and under-5 services, and 4) Systems, including health care financing, supply chain management, health management information system (HMIS), and referral.

### Measures and Data Collection

The impact of EMRI on lives saved was assessed using monthly data on 6 key indicators: 1) antenatal coverage (ANC), 2) skilled birth attendance coverage (SBA), 3) post-natal coverage (PNC), 4) HIV testing during ANC, 5) measles vaccination coverage, and 6) pentavalent 3 vaccination coverage. We also tracked the impact of the EMRI intervention on basic PHCU infrastructure, including access to water and electricity and adherence to recommended staffing levels per FMOH guidelines (Refer to **Appendix S1** for detailed indicator definitions). We tracked PHCU performance monthly on each of these indicators throughout the 18-month intervention period. In addition, we tracked monthly performance for 12 months following the intervention for Phases I and II PHCUs to understand sustained performance levels after the intervention was completed. Data were collected by PHCU staff and corroborated through on-site review of data reports and PHCU registers by the research team every quarter.

### Data Analysis

#### Maternal and child survival

To assess EMRI impact on maternal and child survival, we used Spectrum Policy Modeling System software to determine the number of deaths averted. The Spectrum Policy Modeling System software (Version 4.43) is a suite of 9 policy models prepared by the USAID Health Policy Initiative in collaboration with the Futures Group International. The Spectrum software has been peer-reviewed and is the software used by UNAIDS to prepare annual reports on national and global HIV/AIDS estimates [20].

Three of the models in the Spectrum software were used in conjunction to estimate impacts on mortality at the population level: 1) Lives Saved Tool (LiST): A model to estimate the impact on child and maternal survival following implementation of programs to increase coverage of various health interventions, 2) Demography (DemProj): A model to estimate population projections and demographic indicators over time used simultaneously with LiST in order to provide inputs for calculations of impact on mortality, and 3) AIDS Impact Model (AIM): A model used simultaneously with LiST to simulate the AIDS epidemic and estimate the number of new infections, people living with HIV/AIDS, and AIDS deaths.

The LiST model, which projects changes in maternal and child survival based on changes in the coverage of different child health interventions, requires inputs derived from the data we obtained on the 6 key indicators: ANC, SBA, PNC, HIV testing during ANC, measles vaccination coverage and pentavalent 3 vaccination coverage (Refer to **Appendix S2** for a detailed description of the inputs to the model). To generate estimated performance on the key indicators at each point in time, which underpinned the input values for the LiST model, we employed two-part linear regression for the health centers in each Phase of EMRI. Using separate models for each health indicator, we regressed the health outcome (e.g., ANC care, HIV testing) on time in months, using months 0–18 of the intervention. We repeated this process using data from the 12 months after the intervention (i.e., months 19–30) for Phases I and II. We tested for alternative functional forms but found no significant departure from linear trend, so we employed linear regression. We did find the interaction between phase and time to be significant and, therefore, modelled each phase independently rather than combining data from all phases in a single analysis. Rather than modelling continuous performance change over time, the LiST model requires annual estimates of each indicator. Therefore, we used estimates drawn from the middle of each program year to represent average annual performance (6 months for Year 1, 18 months for Year 2). Because EMRI was 18-months, we attributed only half of the Year 2 estimates lives saved to EMRI. In addition, we used estimated performance at 30 months to approximate post-EMRI (i.e., months 18–30) levels of performance of Phase I and II health centers. Because no follow-up data were available for Phase III health centers, post-EMRI performance was estimated using the 18-month level of performance. We calculated the impact of EMRI by program end, as well as the additional impact if health center performance levels were maintained for an additional five years in the current EMRI catchment areas. Because lessons learned from each phase were incorporated into subsequent interventions, we believe that Phase III represents the most evolved EMRI model. Therefore, we also estimated the total lives that could be saved if the EMRI program were scaled nationally and sustained for 5 years at Phase III performance levels.

### Cost-benefit Ratio of EMRI

We calculated a cost-benefit ratio of the EMRI program including both the lives saved during the 18-month EMRI intervention and a 5-year follow-up period in the catchment areas of the 30 health centers:



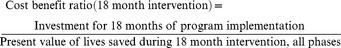


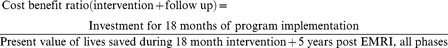



Additionally, we calculated the cost-benefit ratio if evaluation funding were included. We estimated the value of the lives saved assuming 40 disability-free years of life for a child and 20 disability-free years of life for an adult. We selected the approach of valuation of years of lives saved using average GDP per capita per year for valuation, a method applied by the World Bank [21], [22], as a conservative approach to estimating the benefits of the intervention. An alternative approach, the Value of Statistical Life methodology, would aggregate individuals’ willingness to pay to reduce the risks of premature death. Although this method is increasingly being applied in middle- and high-income settings, it has not been extensively used in low-income countries, and data needed for such valuation was not available. We monetized the value using the annual Ethiopian gross domestic product (GDP) per capita ($357) per disability-free year saved, and we used a discount factor of 3% to calculate the present value of lives saved [23]. We then applied the World Health Organization benchmarks [24] by which an intervention that costs less per disability-free life year saved than the GDP per capita per year is considered to be cost effective; this approach is highly conservative in assigning benefits to a saved human life as a person’s ability to generate income.

## Results

### Impact of EMRI on Health Center Infrastructure and Performance

All EMRI health centers experienced improvements in infrastructure. At baseline only 27% of EMRI health centers had access to water; by program end, 100% had access to water (**Table 1**). At baseline, 73% of health centers had access to electricity; by program end, 97% of health centers had access to electricity. Health center staffing increased from 75% of government human resource standards at baseline to 90% by program completion. In addition to these improvements in infrastructure and human resources, several service-related performance indicators showed marked improvements during the 18 months of the EMRI intervention followed by a modest attenuation in performance in the 12 months following the implementation **(** **Table 2** **)**. Despite differences by Phase, improvements were apparent in the proportion of pregnant women who had at least 4 ANC visits, (4 visits per person), the percent of women in ANC care who received an HIV test, skilled birth attendance, PNC care, and the percent of children who received their third pentavalent vaccine **(** **Table 2** **)**.

**Table 1:** Resources in the 30 EMRI^1^ health centers during the 18-month intervention period.

	Percent of health centers with access to electricity		Percent of health centers with access to water		Percent of health care workers assigned to the health center vs. government standards^2^	
	Baseline	Phase out	Baseline	Phase out	Baseline	Phase out
Phase I	70%	100%	30%	90%	55%	85%
Phase II	100%	100%	40%	100%	99%	93%
Phase III	50%	100%	10%	100%	70%	96%
**Total**	73%	100%	27%	97%	75%	90%

1 Ethiopian Millennium Rural Initiative.

2 Calculations for human resources only considered the standard for total clinical staff and did not consider details for each professional category. The government standard for health centers is 16 staff per health center.

**Table 2:** Inputs to the Lives Saved Tool (LiST) Model (Expressed as a %).

	Baseline	Year 1	Year 2	Post intervention
**Phase I**				
Percent of pregnant women who attended 4 or more antenatal care (ANC) visits	20	23	33	23
Percent of pregnant women who received HIV testing during ANC	60	61	87	67
Percent of pregnant women who received skilled birth attendance (SBA)	8	6	9	6
Percent of newborn children who received post-natal care (PNC)	14	41	13	41
Percent of infants under the age of 1 who received measles vaccinationbefore their first birthday	34	72	95	72
Percentage of infants under the age of 1 who received a thirddose of pentavalentvaccine before their first birthday	54	68	100	68
**Phase II**				
Percent of pregnant women who attended 4 or more antenatal care (ANC) visits	19	30	36	37
Percent of pregnant women who received HIV testing during ANC	86	86	87	86
Percent of pregnant women who received skilled birth attendance (SBA)	10	13	15	19
Percent of newborn children who received post-natal care (PNC)	9	18	23	43
Percent of infants under the age of 1 who received measlesvaccination before their first birthday	59	80	91	99
Percentage of infants under the age of 1 who received athird dose of pentavalent vaccine before their first birthday	91	99	100	100
**Phase III**				
Percent of pregnant women who attended 4 or more antenatal care (ANC) visits	24	32	36	N/A
Percent of pregnant women who received HIV testing during ANC	59	84	97	N/A
Percent of pregnant women who received skilled birth attendance (SBA)	8	11	12	N/A
Percent of newborn children who received post-natal care (PNC)	20	33	41	N/A
Percent of infants under the age of 1 who received measlesvaccination before their first birthday	100	100	100	N/A
Percentage of infants under the age of 1 who received athird dose of pentavalent vaccine before their first birthday	100	100	100	N/A

PHCUs demonstrated sustainable performance in the post-intervention period, measured for Phase I and II health centers. Although the performance on some indicators decreased slightly from peak performance during the intervention, the decline was modest, and performance in the year after the intervention exceeded baseline performance for all indicators **(** **Table 2** **)**. Three indicators (PNC, measles and pentavalent 3 vaccination coverage) continued to demonstrate an increasing trend in the year after the EMRI intervention.

### Impact of EMRI on Mortality

An estimated 134 lives (all children) were saved were attributable to the EMRI intervention during the 18-month intervention across the 30 health centers. During the 5-year post-EMRI period, we estimated lives saved at 852 lives (850 children and 2 adults) (**Table 3**). At this performance level and expected population growth, scaling up EMRI for 5 years across the 4 major regions of Ethiopia (Amhara, Oromia, Tigray and SNNPR regions) could save as many as 34,908 lives. Scaling up to the rest of the nation by including the 4 major regions and the emerging regions (Afar, Benishangul- Gumuz, Gambella, and Somali regions) would save an estimated 38,415 lives.

**Table 3:** Maternal and child lives saved in the catchment areas of the 30 health centers.

	18 month EMRI intervention			5 years post-EMRI		
	Maternal	Child	Total	Maternal	Child	Total
Phase I	0	46	46	0	295	295
Phase II	0	49	49	2	393	394
Phase III	0	39	39	0	162	162
**Total**	**0**	**134**	**134**	**2**	**850**	**852**

### Cost-effectiveness of EMRI

For the 18-month intervention period, implementing EMRI cost $37,313 per life saved ($5 million investment and 134 lives saved). If evaluation costs are included, EMRI cost $42,366 per life saved ($5,677,000 per 134 lives saved). The present value of a child’s life saved was calculated as $4,378 (40 years multiplied by $357 (the per capita annual GDP in Ethiopia) per year all divided by the discount factor of (1.03)^40^). Using the World Health Organization benchmarks, an intervention would be deemed cost-effective as long as it costs less than 3 times the present value of the lives saved, estimated using 3 times the nation’s per capita GDP. This value would be $13,161 per life saved ($4,378 times 3, per life saved), which is far less than $37,313 EMRI cost per life saved. The EMRI intervention, assuming only 18 months of improved health center performance, is therefore deemed to be not cost effective.

For the full 5-year follow up period, results differ markedly. Assuming the performance attained during the EMRI intervention was sustained for an additional 5 years, the EMRI would be strongly cost effective, however. The EMRI cost would then be $5,071 per life saved ($5 million investment and a total of 986 lives saved (134 lives from the first 18 months plus an additional 852 in the subsequent 5 years). Counting evaluation costs, the EMRI cost would be $5,758 per life saved ($5,677,000 and 986 lives saved). All but two of the lives saved are of children. The present value of a child’s life saved was calculated as $4,378.63, as above, while the present value of an adult’s life saved was calculated as $3,953.24 (assuming 20 years of disability-free life discounted at 3%). Thus, the discounted value of lives saved is $4,307,590 (calculated as $4,378.63×984). The discounted value of the adult’s lives saved is $7,906 (calculated as $3,953×2), summing to a total value of $4,315,496. Again applying the standard of 3 times the GDP for valuation purposes, the value would be $12,946,488, far exceeding the original investment and hence very cost-effective by World Health Organization standards.

In sensitivity analysis, even if the performance of the health center dropped by more than half its assumed performance levels, which would be below the baseline performance, the 18-months of EMRI implementation would be deemed cost-effective using World Health Organization benchmarks. Alternatively, the program could more than double its investment over the 5 years following the original investment, and it would be deemed cost effective.

## Discussion

In this comprehensive evaluation of the EMRI intervention, we found transformational impacts on multiple areas including the health system infrastructure, human resource capacity and utilization of HIV treatment services, as well as cost-effectiveness in terms of lives saved over 5 years of follow up. The investments in critical infrastructure such as access to water and electricity and improvements to buildings will have far-reaching benefits for health care delivery in rural settings. Strengthening of human resources, particularly developing management, included training managers to advocate with the *woreda* health offices to support the health centers and health extension workers effectively. Management advocacy and support of front-line providers is key to continued development of this level of the delivery system to improve rural health care. In addition, EMRI expanded care and treatment services for HIV. HIV testing was implemented at the health post level by health extension workers, an important shifting of tasks in order to increase access. HIV treatment protocols and clinical mentoring were established to enable HAART at nucleus health centers (previously prohibited and now promoted as part of government policy).

The analysis underscores the particular importance of sustained performance in driving cost effectiveness. Although the pay back in terms of lives saved did not confer cost effectiveness in the first 18 months, with 5 years of sustained performance, the benefits far outweigh the costs of the program. Even if the performance of health centers diminished somewhat after the first 18 months, which is unlikely given the continued strong performance of the 20 health centers monitored for a year after the 18-month intervention period, the EMRI investment would still be deemed cost effective by World Heath Organization benchmarks. Because the EMRI applied a systems-based approach, added resources after the first 18 months were minimal; however, performance largely sustained and lives saved accelerated, resulting in increasing cost-effectiveness over time. Although additional resources would need to be committed to scale up the EMRI throughout the major regions of Ethiopia and across the country, given the experience within the 30 original *woredas,* such an investment would pay for itself in terms of lives saved, particularly children, in the country. Importantly, the EMRI was designed in close collaboration with Ministry of Health, and national scale up was part of the original vision. The program was hence designed to embed in national reform efforts related to primary care without substantial increased investment.

Several aspects of EMRI were central to its success. The PMTCT Fast Track, a structured approach to problem solving adopted by health center managers and staff, helped staff and clinicians improve adherence to PMTCT guidelines across the health centers and is easily adaptable to transfer to other sites. In addition, the intensive mentoring model built into EMRI involved placing both management and clinical mentors in every health center for 6–12 months. This close and continued mentorship allowed for supportive supervision in a range of areas, to identify and address challenges to program implementation in real-time. Finally, the intentional investments in system-wide improvements (such with supply chains and laboratories) made during EMRI have strengthened the horizontal capacity of the rural health care system, of benefit to patients and families well beyond provision of select services for the treatment of targeted diseases.

Our results should be interpreted in light of several limitations. First, the LiST model likely underestimates the health benefits of EMRI. Predictions are affected by changes in performance in only six of the EMRI indicators, and many infrastructure improvements (e.g., the percentage of health centers with water or recommended staffing levels) do not translate into direct model inputs to the LiST model that estimates lives saved. Hence, our estimates of impact may be overly conservative. Second, although we followed PHCUs for 12 months after the completion of the EMRI intervention to understand potential declines in performance, we were unable to follow PHCUs for 5 years to have empirical data on longer run performance. Last, we used the current annual GDP to monetize the disability life-years saved, given the pace of economic development in Ethiopia, this likely underestimates the benefits substantially, again resulting in a conservative estimation of the impact of EMRI.

Evidence regarding the national impact at scale of a system-based intervention to improve primary health care in rural settings is limited. We found EMRI resulted in a range of positive impacts. Estimation of lives saved is a critical planning tool for decision-makers as they consider allocation of limited resources in the context of many pressing competing demands. Nevertheless, for interventions such as complex, system-level initiatives, lives saved models may not adequately capture the effects of other essential improvements to the system. The implication of our results is that health systems strengthening is imperative, even if the benefits cannot be immediately quantified. Key to sustainability of the gains from systems-level interventions such as EMRI are collaborative, multi-sectoral partnerships such as those forged to design, implement and evaluate EMRI, as well as deliberate attention to develop leadership capacity at all levels of the system. Insights from this evaluation may be useful in informing strategic planning regarding the scale up of this model nationally, as the country continues to apply this approach to building primary care in their 5-year health systems development plan.

## Supporting Information

Appendix S1 Definitions of the 9 indicators used to measure Primary Health Care Unit (PHCU) performance.
(DOC)

Appendix S2 Descriptors of Inputs to the Model.
(DOC)

## References

[1] WHO (1978) Declaration of Alma-Ata. World Health Organization.

[2] Reerink IH, Sauerborn R (1996) Quality of primary health care in developing countries: recent experiences and future directions. Int J Qual Health Care 8: 131–139. 8792168 10.1093/intqhc/8.2.131

[3] Bhutta ZA, Darmstadt GL, Haws RA, Yakoob MY, Lawn JE (2009) Delivering interventions to reduce the global burden of stillbirths: improving service supply and community demand. BMC Pregnancy Childbirth 9 Suppl 1S7. 19426470 10.1186/1471-2393-9-S1-S7 PMC2679413

[4] Delva W, Yard E, Luchters S, Chersich MF, Muigai E, et al (2010) A Safe Motherhood project in Kenya: assessment of antenatal attendance, service provision and implications for PMTCT. Trop Med Int Health 15: 584–591. 20230571 10.1111/j.1365-3156.2010.02499.x

[5] Otchere SA, Kayo A (2007) The challenges of improving emergency obstetric care in two rural districts in Mali. Int J Gynaecol Obstet 99: 173–182. 17904144 10.1016/j.ijgo.2007.07.004

[6] Pronyk PM, Muniz M, Nemser B, Somers MA, McClellan L, et al (2012) The effect of an integrated multisector model for achieving the Millennium Development Goals and improving child survival in rural sub-Saharan Africa: a non-randomised controlled assessment. Lancet 379: 2179–2188. 22572602 10.1016/S0140-6736(12)60207-4

[7] Atherton F, Mbekem G, Nyalusi I (1999) Improving service quality: experience from the Tanzania Family Health Project. Int J Qual Health Care 11: 353–356. 10501606 10.1093/intqhc/11.4.353

[8] Hounton S, Byass P, Brahima B (2009) Towards reduction of maternal and perinatal mortality in rural Burkina Faso: communities are not empty vessels. Glob Health Action 2. 10.3402/gha.v2i0.1947 PMC2779943 20027267

[9] Brazier E, Andrzejewski C, Perkins ME, Themmen EM, Knight RJ, et al (2009) Improving poor women’s access to maternity care: Findings from a primary care intervention in Burkina Faso. Soc Sci Med 69: 682–690. 19596165 10.1016/j.socscimed.2009.06.023

[10] Mushi D, Mpembeni R, Jahn A (2010) Effectiveness of community based Safe Motherhood promoters in improving the utilization of obstetric care. The case of Mtwara Rural District in Tanzania. BMC Pregnancy Childbirth 10: 14. 20359341 10.1186/1471-2393-10-14 PMC2858713

[11] Bancheno WM, Mwanyumba F, Mareverwa J (2010) Outcomes and challenges of scaling up comprehensive PMTCT services in rural Swaziland, Southern Africa. AIDS Care 22: 1130–1135. 20824565 10.1080/09540121003615079

[12] Solomon Y, Ballif-Spanvill B, Ward C, Fuhriman A, Widdision-Jones K (2008) The dynamics of community and NGO partnership: primary health care experiences in rural Mali. Promot Educ 15: 32–37. 19066236 10.1177/1025382308097696

[13] Arifeen SE, Hoque DM, Akter T, Rahman M, Hoque ME, et al (2009) Effect of the Integrated Management of Childhood Illness strategy on childhood mortality and nutrition in a rural area in Bangladesh: a cluster randomised trial. Lancet 374: 393–403. 19647607 10.1016/S0140-6736(09)60828-X

[14] Ginsburg AS, Hoblitzelle CW, Sripipatana TL, Wilfert CM (2007) Provision of care following prevention of mother-to-child HIV transmission services in resource-limited settings. AIDS 21: 2529–2532. 18025890 10.1097/QAD.0b013e3282f155f4

[15] Cowan FM, Pascoe SJ, Langhaug LF, Mavhu W, Chidiya S, et al (2010) The Regai Dzive Shiri project: results of a randomized trial of an HIV prevention intervention for youth. AIDS 24: 2541–2552. 20881473 10.1097/QAD.0b013e32833e77c9 PMC3058934

[16] Ahluwalia IB, Robinson D, Vallely L, Gieseker KE, Kabakama A (2010) Sustainability of community-capacity to promote safer motherhood in northwestern Tanzania: what remains? Glob Health Promot 17: 39–49. 20357351 10.1177/1757975909356627

[17] Bradley E, Thompson JW, Byam P, Webster TR, Zerihun A, et al (2011) Access and quality of rural healthcare: Ethiopian Millennium Rural Initiative. Int J Qual Health Care 23: 222–230. 21467077 10.1093/intqhc/mzr013

[18] Bradley EH, Byam P, Alpern R, Thompson JW, Zerihun A, et al (2012) A systems approach to improving rural care in Ethiopia. PLoS One 7: e35042. 22558113 10.1371/journal.pone.0035042 PMC3338815

[19] Curry LA, Alpern R, Webster TR, Byam P, Zerihun A, et al (2012) Community perspectives on roles and responsibilities for strengthening primary health care in rural Ethiopia. Glob Public Health 7: 961–973. 22621744 10.1080/17441692.2012.686114

[20] Stover J, Johnson P, Zaba B, Zwahlen M, Dabis F, et al (2008) The Spectrum projection package: improvements in estimating mortality, ART needs, PMTCT impact and uncertainty bounds. Sex Transm Infect 84 Suppl 1i24–i30. 18647862 10.1136/sti.2008.029868 PMC2569834

[21] Ashenfelter O (2006) Measuring the value of a statistical life problems and prospects. Discussion paper no 1911. Bonn, Germany: IZA,.

[22] He J WH (2010) The value of Statistical Life: A contigent investigation in China. World Bank Policy Research Working Paper 5421.

[23] Bank W (2011) Gross Domestic Product (GDP) per capita (current US$). World Bank.

[24] Organization WH (2013) CHOosing Interventions that are Cost Effective (WHO-CHOICE): Table: Threshold vlues for intervention cost-effectiveness by Region.

