# Supplementary material for: Evaluation of the Ethiopian Millennium Rural Initiative: Impact on Mortality and Cost-Effectiveness
Source: PLoS One. 2013 Nov 18;8(11):e79847. doi: 10.1371/journal.pone.0079847 (PMC3832618; doi:10.1371/journal.pone.0079847)
Supplement: Appendix S1 — Definitions of the 9 indicators used to measure Primary Health Care Unit (PHCU) performance. (DOC) [file pone.0079847.s001.doc]

**Appendix S1. Definitions of the 9 indicators used to measure Primary Health Care Unit (PHCU) performance**

| **Indicator** | **Definition** |
| --- | --- |
| Antenatal coverage (ANC)* | ANC visits were calculated as the number of women who initiate ANC divided by the expected number of pregnancies in the catchment area, based on national epidemiologic rates. |
| Skilled Birth Attendance (SBA)* | SBA was calculated as the number of women who give birth in the health center divided by the expected number of births in the catchment area given national epidemiologic rates. |
| Post Natal Care (PNC)* | PNC was calculated as the number of women who sought postnatal care divided by the expected number of births in the catchment area given national epidemiologic rates. |
| HIV testing during among pregnant women* | HIV testing among pregnant women during their first ANC visit was calculated as the number of HIV tests provided to pregnant women divided by the number of women seen in their first ANC visit. |
| Measles coverage* | Measles vaccination coverage was calculated as the number of infants who received a dose of the measles vaccine before their first birthday divided by the expected number of infants under the age of 1 in the catchment area given national epidemiologic rates. |
| Pentavalent 3 coverage* | Pentavalent 3 vaccination coverage was calculated as the number of children who received a third dose of pentavalent vaccine before their first birthday divided by the expected number of infants under the age of 1 in the catchment area given epidemiologic rates |
| Access to water | The number of health centers with access to a water source divided by the total number of health centers |
| Access to electricity | The number of health centers with access to electricity divided by the total number of health centers |
| Adherence to staffing standards | The number of health care professionals divided by the recommended number of health care professionals based on Ethiopian Federal Ministry of Health (FMOH) standards |

*Indicators used as inputs to the Lives Saved Tool (LiST) model

**Appendix S2. Descriptors of Inputs to the Model**

ANC coverage

The ANC coverage level in the LiST model was defined in the model as the percentage of women with at least four ANC visits. Because EMRI indicator data report the percentage of women with at least one ANC visit, we adjusted the EMRI indicator data in order to fit the model requirements. Although EMRI also made improvements in the number of women with less than four ANC visits, this will be an underestimation of the impact of increased ANC services since the LiST model does not accommodate mortality estimation based on these data.

We assumed that 33% of women who have at least one ANC visit (as measured by EMRI indicators) would in fact have at least four ANC visits (as specified in the LiST model). We made this assumption based on the distribution of data in the Ethiopia 2005 national DHS data for the number of ANC visits by pregnant women in rural areas 59 [0 (75%), 1 (5%), 2-3 (11%), 4+ (8%)]10. Therefore we substituted EMRI data on ANC coverage for the LiST model inputs, adjusted to estimate the percentage of women with at least four ANC visits.[[1]](#footnote-2)

We allowed Spectrum to calculate the coverage for some components that comprise antenatal care and facility based birth interventions, and we made assumptions for other components. We assumed that 4 ANC visits would include 50% of ANC coverage levels of case management interventions (MgSO4 management of pre-eclampsia, hypertensive disease management, and malaria management at the clinic and hospital level) and 50% of ANC coverage of supplementation interventions when needed (calcium, multiple micronutrient, balanced energy)[[2]](#footnote-3). The model specified 20% of the ANC coverage level for syphilis detection and treatment. Because coverage levels for tetanus toxoid are already high (84%), we did not simulate an impact on this coverage level due to EMRI increase in ANC services. Child deaths prevented by increasing ANC coverage were a result of short- and long-term effects and included reductions in neonatal diarrhea, neonatal asphyxia, neonatal sepsis pneumonia, diarrhea, pneumonia, measles, and malaria. Maternal deaths prevented by increasing ANC coverage were a result of reductions in malaria and hypertensive diseases in pregnancy.

Skilled delivery coverage

The LiST model allows separate inputs for child birth coverage for institutional (clinic and hospital) deliveries and overall skilled birth attendance (such that allowances can be made for skilled birth attendance occurring outside of a clinic or hospital setting), with the remaining child births categorized as unassisted deliveries. We used the EMRI skilled delivery coverage for both the institutional delivery and overall skilled birth attendance, as we have no information on the number of additional skilled births occurring outside of the health centers and health posts. Therefore we did not include the impact of neonatal resuscitation or clean practices and immediate essential newborn care which occurred in the home or health post.

We allowed the Spectrum model to calculate the coverage for the majority of components that comprise institutional and home delivery. Spectrum calculated that 90% of skilled deliveries would receive essential obstetric care, and 10% would receive comprehensive emergency obstetric care. Of those receiving essential obstetric care, Spectrum estimated that 100% would receive clean birth practices, immediate assessment and stimulation, and labor and delivery management. We specified varying levels of associated service coverage for essential obstetric care: 20% of the institutional coverage level for antenatal corticosteroids for preterm labor and neonatal resuscitation, and 50% of the institutional coverage level for antibiotics for premature rupture of membranes, active management of the 3rd stage of labor, and management of eclampsia with MgSO4[[3]](#footnote-4) We allowed Spectrum to calculate the coverage levels of the same components for women receiving comprehensive emergency obstetric care.

In the model, changes in skilled delivery coverage result in changes in cause-specific neonatal and maternal mortality rates, which are subsequently translated into lives saved. Child deaths prevented by increasing skilled delivery coverage were a result of reductions in neonatal asphyxia, neonatal sepsis and pneumonia, neonatal tetanus, and prematurity. Maternal deaths prevented by increasing skilled delivery coverage were a result of reductions in antepartum hemorrhage, postpartum hemorrhage, hypertensive diseases of pregnancy, sepsis, and obstructed labor.

Post-natal coverage

EMRI post-natal care coverage was measured as the percentage of woman coming for at least one post-natal visit. Post-natal care was not a single input in the LiST model but rather a series of 27 different inputs (e.g., breastfeeding, vitamins, hygiene, treatment of infections, etc.). Based on discussions with EMRI staff regarding the content of post-natal care visits as defined by the FMOH, we chose 5 of these categories for model inputs to represent improvements in EMRI post-natal coverage:

1. Preventive postnatal care. The model stipulates that this is the percentage of infants with a postnatal health contact/visit within two days of birth, whereas the EMRI indicator data collected reflects the percentage of infants with a postnatal visit within 42 days of birth. Based on discussions regarding clinic practices at EMRI health centers, in which post-natal care visits are encouraged on a ‘6-6-6’ plan (at 6 hours, 6 days, and 6 weeks post-delivery), we assumed that one-third of EMRI improvements in post-natal care would be reflected in the ’preventive postnatal care’ parameter, which includes thermal care and clean postnatal practices.

2. Kangaroo mother care, which is the percentage of low birth weight infants receiving skin-to-skin contact between a mother and her newborn and frequent and exclusive breastfeeding. In discussions regarding clinic practices at EMRI health centers, we found that kangaroo care counseling is consistently provided for premature infants. We therefore used full EMRI improvements in post-natal care for this parameter.

3. Breastfeeding improvement, which is the percentage of mothers counseled to exclusively breastfeed for 6 months post-delivery. Based on discussions regarding clinic practices at EMRI health centers, in which breastfeeding counseling is provided at both ANC and post-natal care visits, we used full EMRI improvements in post-natal care for this parameter.

4. Complementary feeding education, which is the percentage of mothers counseled on the importance of continued breast-feeding after 6 months and on appropriate complementary feeding practices. Again, we found that this counseling is generally part of post-natal care visits in EMRI health centers and therefore used full improvements in EMRI post-natal care for this parameter.

5. Case management of severe neonatal infection is part of the curative postnatal care package. This component is the sum of the percentage of children receiving oral antibiotics, injectable antibiotics and “full supportive care”, which includes treatments like oxygen, IV antibiotics, IV fluids, blood transfusions, and other therapies as necessary. We assumed that 50% of neonates with severe infections were brought in for a post-natal care visit *and* treated with appropriate oral antibiotics, so we assumed 50% of EMRI improvements in post-natal care would be reflected the oral antibiotics parameter. We allowed Spectrum to estimate full supportive care coverage (which would require referral to a hospital) at 0.5% of neonates. We did not include the impact of injectable antibiotics as part of the case management of severe neonatal infection.

We did not change the other input values for post-natal coverage which included interventions involving improved water sources, improvements in hygiene, vitamins for disease prevention, malaria prevention, sepsis management, and treatment of childhood diseases.

Child deaths prevented by increasing post-natal coverage in these simulations were a result of reductions in neonatal diarrhea, neonatal sepsis pneumonia, neonatal asphyxia, prematurity, diarrhea, pneumonia, measles, and malaria. EMRI does not directly address these issues, except through post-natal care coverage. Increasing post-natal coverage did not contribute to the prevention of maternal deaths for the services examined in this study.

Vaccination coverage

The LiST model allows for the estimation of impact on childhood mortality for vaccination coverage with 5 major vaccines (Rotavirus, Measles, Haemophilus influenzae type B (HiB), Pneumococcal, and Diptheria/Pertussis/Tetanus). EMRI indicator data was available for measles and pentavalent vaccination coverage. The pentavalent vaccine consists of HiB, diptheria, pertussis, tetanus, and Hepatitis B. Therefore, we were able to estimate the impact of improvements in levels of measles, HiB, and DPT vaccination, but we could not estimate the impact of improvements in pneumococcal and rotavirus vaccination.

We applied these pentavalent vaccination coverage levels to both the HiB and the DPT inputs in the model. Child deaths prevented by increasing measles, HiB and DPT vaccination coverage were a result of reductions in pneumonia and measles. The LiST model also allows for the simulation of coverage with Bacille Calmette Guerin (BCG) and polio vaccines, but does not allow for estimation of the mortality impact as a result of decreasing or increasing the coverage of these two vaccines.

The AIM model, used in conjunction with the LiST model, allows for the estimation of the impact of increasing ARV prophylaxis in pregnant women. The AIM model requires the percentage of *all* HIV-positive pregnant women receiving ARVs, while the EMRI indicator data reflect the percentage of HIV-positive pregnant women *accessing ANC* who are receiving ARVs. Therefore we adjusted the EMRI indicator data for this parameter to estimate the percentage of all HIV-positive pregnant women who are receiving ARVs[[4]](#footnote-5). To do this, however, we had to assume that there were no differences in HIV infection rates between women who did and did not access ANC services.

We included the percentage of adults and children receiving ARVs and the percentage of children receiving cotrimoxazole at the suggestion of Futures Institute staff, though it should be noted that the Spectrum model does not allow for the differentiation of infant treatment from treatment for all children aged 0-15 years. Accordingly, we are unable to include the impact of EMRI increases in treatment of HIV-exposed infants.

1. Based on this national distribution of data for pregnant women in rural areas, 8% of the population had at least four ANC visits and 24% (5% + 11% + 8%) had at least one ANC visit. Therefore, we estimated that 1/3 (8%/24%) of the pregnant women who had at least one ANC visit in fact had at least four ANC visits. [↑](#footnote-ref-2)
2. This assumption is mostly based on discussions with EMRI staff regarding the content of ANC visits: only ~50% of the HCs were able to refer to a major hospital within one hour’s travel distance; not all clinics had stock of essential medications and supplies; 4 ANC visits does not guarantee a pregnant woman comes in when she actually has pre-eclampsia or malaria; and, this aligns with the model’s assumption that 4 ANC visits only guarantees 20% of syphilis detection and treatment. [↑](#footnote-ref-3)
3. This assumption is based on discussions with EMRI staff regarding the content of ANC visits, as indicated above for pre-eclampsia and malaria treatment assumptions. [↑](#footnote-ref-4)
4. We assumed (probability of pregnant women accessing ANC)x(probability of receiving ARVs for HIV-positive women accessing ANC)=probability of receiving ARVs for pregnant HIV-positive women [↑](#footnote-ref-5)
